# Numerical simulations of atmospheric dispersion of iodine-131 by different models

**DOI:** 10.1371/journal.pone.0172312

**Published:** 2017-02-16

**Authors:** Ádám Leelőssy, Róbert Mészáros, Attila Kovács, István Lagzi, Tibor Kovács

**Affiliations:** 1 Department of Meteorology, Eötvös Loránd University, Budapest, Hungary; 2 Institute of Physics, Budapest University of Technology and Economics, Budapest, Hungary; 3 Institute of Radiochemistry and Radioecology, University of Pannonia, Veszprém, Hungary; Universidade de Vigo, SPAIN

## Abstract

Nowadays, several dispersion models are available to simulate the transport processes of air pollutants and toxic substances including radionuclides in the atmosphere. Reliability of atmospheric transport models has been demonstrated in several recent cases from local to global scale; however, very few actual emission data are available to evaluate model results in real-life cases. In this study, the atmospheric dispersion of ^131^I emitted to the atmosphere during an industrial process was simulated with different models, namely the WRF-Chem Eulerian online coupled model and the HYSPLIT and the RAPTOR Lagrangian models. Although only limited data of ^131^I detections has been available, the accuracy of modeled plume direction could be evaluated in complex late autumn weather situations. For the studied cases, the general reliability of models has been demonstrated. However, serious uncertainties arise related to low level inversions, above all in case of an emission event on 4 November 2011, when an important wind shear caused a significant difference between simulated and real transport directions. Results underline the importance of prudent interpretation of dispersion model results and the identification of weather conditions with a potential to cause large model errors.

## Introduction

In October and November 2011, increased ^131^I concentrations were observed at several European stations, mainly in Central Europe. Stations located in the Czech Republic, Austria, Germany, Poland, Slovakia, Sweden, France, Hungary, Ukraine and Russia have measured and informed the Incident and Emergency Centre (IEC) on the detection of ^131^I in air samples collected over intervals of several days [1–3]. Measured concentration values in some European countries just reached the limit of detection and were not any health concern to the population. However, only a few months after the accident in Fukushima Dai-ichi Nuclear Power Plant in March 2011, these detections received more attention both from the public and the scientific communities [4]. Although the measured concentrations were below the exposure limit, the International Atomic Energy Agency (IAEA) has initiated an investigation in order to find out the source of the increased iodine concentrations. Backward trajectory simulations narrowed the possible locations of the source to Central Europe, however, they could not perform more accurate localization because of the long sampling period of measurement sites [3]. The investigation led to result on 17 November 2011, when the Hungarian Atomic Energy Authority reported that during the period between January and May, and also between September and November, 2011, probably due to the improper operation of the filtration system, some ^131^I, slightly higher than usual, had been released into the atmosphere from the laboratory of the Institute of Isotopes Ltd., Budapest [5]. The Institute of Isotopes is dealing with the research, development and production of a wide variety of radioactive isotopes and other products for a broad range of application areas, especially healthcare, research and industry.

In this study, the dispersion of the radioactive plume and the spatial distribution of ^131^I were simulated by different dispersion models over Central Europe. The applied models were the following:

- the HYSPLIT (Hybrid Single-Particle Lagrangian Integrated Trajectory) model of the National Oceanic and Atmospheric Administration (NOAA),
- the RAPTOR Lagrangian dispersion model, developed by the authors at Eötvös Loránd University (ELU),
- and the WRF-Chem Eulerian integrated atmospheric chemistry transport model (WRF-ARW modell—*The Weather Research & Forecasting Model—Advanced Research WRF*, v3.6, 2014 coupled with *WRF-Chemistry*, v3.6, 2014).

The intercomparison of the results of different dispersion models were used to evaluate model performance and point out uncertainties in a complex weather situation in Central Europe on 4 November 2011.

The ^131^I emission timeline has been reconstructed by the Institute of Isotopes. On the other hand, unfortunately, only very few measurement data have become publicly available after the incident. By the intercomparison of emission events and reported detections [2,3,5], supported by atmospheric trajectories, four source-receptor connections could be identified:

- an emission event on 17 October 2011 caused the increase of atmospheric aerosol ^131^I concentration to 19.2 μBq/m^3^ in Dubna, Russia on 20–21 October;
- plume from a release on 24 October 2011 was detected in Prague, Czech Republic with concentrations in the magnitude of 1 μBq/m^3^;
- emission on 31 October 2011 caused elevated concentrations in Stockholm, Sweden on 3–5 November, reaching 5.6 μBq/m^3^;
- emission on 4 November 2011 was detected in Prague with concentrations in the magnitude of 10 μBq/m^3^.

Emission rate was assumed to be uniform between 9–15 UTC on each release day according to operational processes of the factory. Daily total emitted ^131^I activity on the four investigated days was 25.0, 9.2, 16.3 and 14.8 GBq, respectively.

The evaluation of dispersion models investigates two important aspects: whether and when the predicted plume reached the detection site (arrival time), and what maximum concentration it reached [6–8]. Arrival times and affected areas are usually well predictable with dispersion models, while peak values of concentrations show larger uncertainty. The accuracy of the model largely depends on the complexity of the weather situation and the chemical interactions of the pollutants. In archive dispersion studies, analysis and short-range forecast meteorological data is used as the input of the dispersion simulation. These datasets usually have small forecast error, therefore, weather-related uncertainty is mostly due to unrepresented subgrid scale phenomena of convection, low-level inversion, orographic effects and local winds. From the four investigated releases, the one on 4 November 2011 occurred in a complex weather situation where orography and a low-level inversion with wind shear played a significant role in the atmospheric transport of the emitted radioiodine. The investigation of model performance in this hardly predictable situation is the main subject of this study.

## Model description

The study uses two Lagrangian dispersion models, the HYSPLIT model, one of the most widely used software for atmospheric dispersion simulations [9,10]; and RAPTOR, a Lagrangian model developed at Eötvös Loránd University [6,11]. Lagrangian models calculate atmospheric trajectories of particles originating from the source. The particle motion is defined as the superposition of a deterministic downwind term (advection) and a stochastic turbulent motion [9,12]. The deterministic term is interpolated to each particle position from the wind field provided by an input numerical weather prognostic (NWP) model. Input meteorological data is obtained from analysis or forecast fields of the GDAS/GFS model (Global Data Assimilation System, Global Forecast System). While the deterministic downwind motion largely defines the direction of the transport of the plume, turbulent diffusion is responsible for horizontal and vertical mixing. Turbulence efficiency is calculated from planetary boundary layer (PBL) parameterizations based on the Monin—Obukhov length as a stability parameter. In HYSPLIT, a vertical eddy diffusivity (*K*_z_) based approach is applied [9], while RAPTOR uses Hanna's method to calculate turbulent velocity fluctuations in three directions [13,14]. The common principle of both approaches is that they use a pre-calculated PBL height, and turbulence intensity is given as a universal vertical profile function between the surface and the top of the PBL [13]. Therefore, PBL height is a key parameter of the dispersion model that largely influences the results. It is especially true if there is a significant wind shear, either horizontal or vertical, thus relatively small errors in the location of the plume can result in significantly different downwind transport directions. PBL height is either calculated from potential temperature stratification [9] or obtained as input data from the NWP model.

In our study, HYSPLIT has been treated as a reference model, being one of the most widely applied and evaluated dispersion models. It was extensively applied for research and decision support in case of the Fukushima accident and the Eyjafjallajökull volcano eruption, as well as several worldwide investigations of regional to local scale dust and air pollution transport [10,15–17]. The models were used with GDAS/GFS (Global Data Assimilation System/Global Forecast System) meteorological data with 0.5 degree resolution. GFS data was obtained from the NOMADS (National Operational Model Archive and Distribution System) data center [18].

Transport of particles towards Stockholm and Dubna was modeled with 84-hour simulation time, while the two cases in Prague were simulated with 24-hour long simulation runs. The number of released particles was set to 1,000,000 for the short-range and 2,000,000 for the long-range simulations in both models. Concentration was calculated on a rectangular grid with 0.1-degree resolution. The vertical depth of the averaged layer on the surface was chosen to be 100 m and the dry deposition velocity was set to 0.4 cm/s, this value was used in HYSPLIT simulations of radioiodine aerosol originating from Fukushima [15]. Radioactive decay was also taken into account in the models. Measurements detected only the aerosol fraction of iodine. Unfortunately, there is no information on the initial ratio of gaseous to aerosol ^131^I in the emission, therefore all the emitted amount was considered to be in aerosol form.

For the short-range simulations, the online integrated atmospheric chemistry transport model WRF-Chem was also used to compare the results obtained from two different types (Lagrangian and Eulerian) of models. WRF-Chem is an open-source model developed at NOAA [19,20]. It performs the simulation of weather and dispersion in an online coupled way with fine spatial and temporal resolution. The WRF-Chem model has been widely applied in the simulation of air quality as well as the dispersion of radioactive pollutants [21–25]. Although the finer resolution and the coupling of meteorological and chemical processes provide an increased accuracy, WRF-Chem requires significantly larger computational capacities than Lagrangian models that limits its application in fast-response decision support models. Due to the large computational cost, WRF-Chem was only applied in the short-range simulations of the detections in Prague.

In this study, the WRF-Chem model domain consisted of 16,000 grid points and 39 vertical levels covering Central Europe between the 9–29°E longitudes and the 43–52°N latitudes. The horizontal resolution was set to 10 km. Boundary conditions were provided by the GFS model with 0.5-degree resolution. Radioiodine was simulated as a passive tracer taking into account dry deposition and radioactive decay. Planetary boundary layer turbulence was calculated with the Mellor—Yamada—Janjic parameterization [26]. Some experiments have been also made with different PBL schemes (namely ACM2 and YSU) without significant impact on model results.

## Results

**Fig 1:**
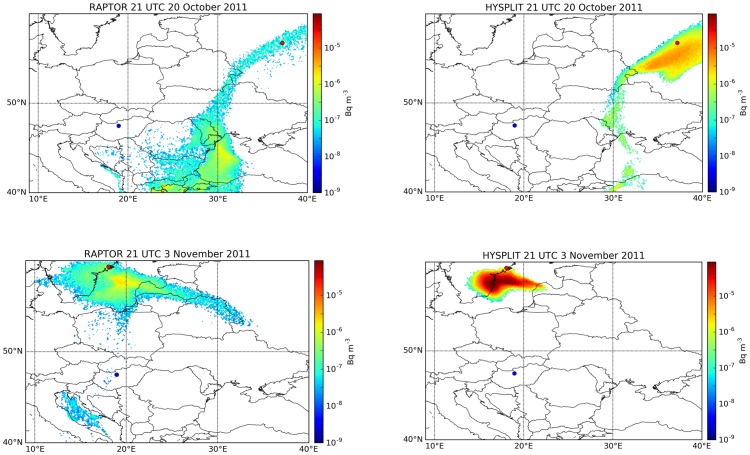
Modeled surface concentration of ^131^I 84 hours after the beginning of emission. Top: 21 UTC, 20 October 2011; bottom: 21 UTC, 3 November 2011; left: RAPTOR model results; right: HYSPLIT model results. Blue dot: source location (Budapest). Red dots: detection sites (Dubna and Stockholm).

Long-range simulations were performed with HYSPLIT and RAPTOR Lagrangian models to investigate the prediction of detections in Dubna (Russia) and Stockholm (Sweden), located in a distance of 1600 and 1300 km from the emission source. The release days were 17 and 31 October, respectively. The transport direction was correctly predicted in both models, however, the altitude of particles and therefore the surface concentrations showed large uncertainty (Fig 1). RAPTOR predicted peak concentrations of 0.1 μBq/m^3^ in Dubna and 0.3 μBq/m^3^ in Stockholm, which were significantly lower than measured values (19.2 and 5.6 μBq/m^3^ in Dubna and Stockholm, respectively). At the same time, the arrival times were correctly predicted by RAPTOR as 9 UTC, 20 October and 19 UTC, 3 November, respectively, both times being within the sampling period of the reported detections.

The surface concentration values predicted by HYSPLIT were generally larger and reached 1.6 and 51 μBq/m^3^ on the surface in Dubna and Stockholm, respectively (Fig 1). The plume of HYSPLIT was more concentrated in both cases, affecting a smaller area with larger concentrations than in case of the RAPTOR simulation.

The weather in Dubna was characterized by weak rain and fog during the sampling period. Precipitation was also present to the south of Stockholm during the sampling period, and fog was reported from the city, indicating a similar weather condition where low-level temperature inversion capped the near-surface pollutants. Although the complex weather situation caused large uncertainties in surface concentration values, both models correctly predicted the presence of the plume at the two detection sites. It can be concluded that continental scale dispersion is largely dominated by the more certain large-scale wind characteristics and uncertainty mainly lies in the vertical mixing above a specific receptor point [27].

**Fig 2:**
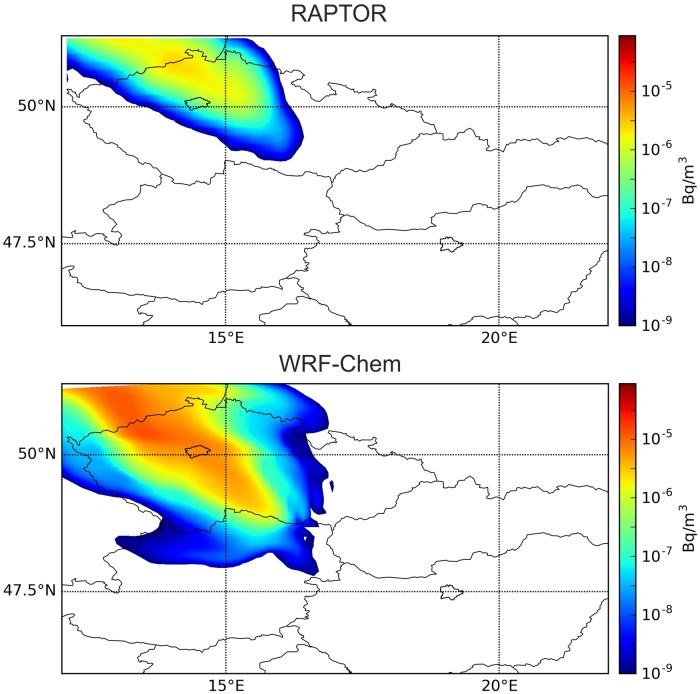
Reported detection in Prague on 24–25 October was correctly predicted by the models. Modeled surface concentration of ^131^I at 0 UTC, 25 October 2011 with a Lagrangian (RAPTOR) and an online integrated Eulerian model (WRF-Chem) using the WRF meteorological data. The cities of Prague and Budapest are outlined.

**Fig 3:**
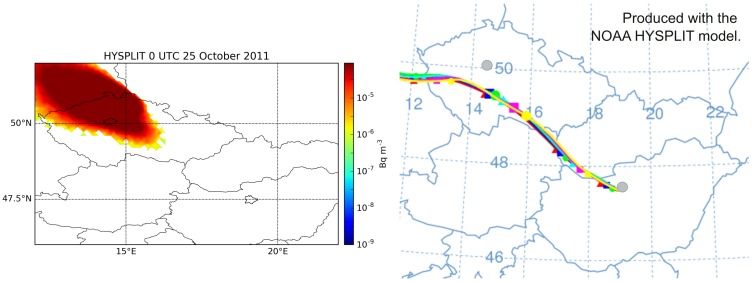
HYSPLIT model results for 25 October 2011. Left: surface concentration of ^131^I at 0 UTC, 25 October 2011; right: 24-hour trajectories started hourly between 9–15 UTC, 24 November 2011. The locations of Prague and Budapest are marked. Simulated with the HYSPLIT model [10] using the GFS meteorological data.

Atmospheric ^131^I aerosol in the activity magnitude of 1 μBq/m^3^ was detected between 24 and 26 October 2011 in Prague, Czech Republic, in a distance of 450 km to the north-western direction from the source location [2]. RAPTOR model simulation based on GFS (Global Forecast System, [18]) meteorological data was carried out evaluating 1,000,000 single particle trajectories. The results were compared to the results from WRF-Chem Eulerian and HYSPLIT Lagrangian models. All simulation results correctly predicted the presence of the plume in Prague (Figs 2 and 3). Predicted arrival times were similar in all three models, ranging between 16–20 UTC, 24 October 2011. Peak concentration estimates, however, showed a large uncertainty with values ranging several magnitudes from 0.6 μBq/m^3^ (RAPTOR) through 65 μBq/m^3^ (WRF-Chem) to 267 μBq/m^3^ (HYSPLIT).

**Fig 4:**
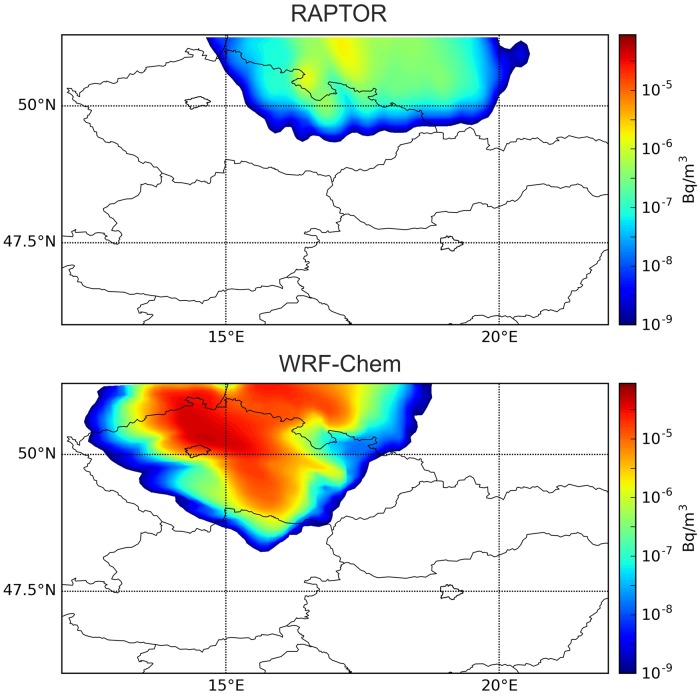
Reported detection in Prague on 4–5 November was correctly predicted only by the WRF-Chem model. Modeled surface concentration of ^131^I at 0 UTC, 5 November 2011 with a Lagrangian (RAPTOR) and an online integrated Eulerian model (WRF-Chem) using the WRF meteorological data. The cities of Prague and Budapest are outlined.

**Fig 5:**
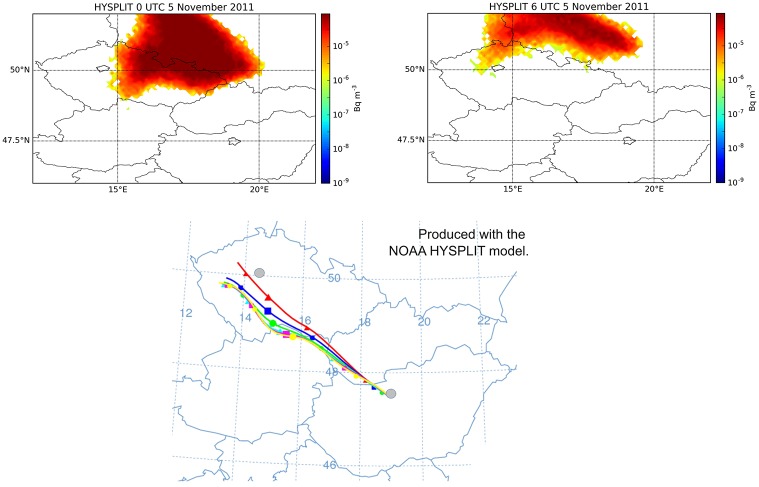
The contradiction between dispersion and trajectory results can be explained with the large vertical wind shear. Top: surface concentration of ^131^I at 0 and 6 UTC, 5 November 2011; bottom: 24-hour trajectories started from 40 m height hourly between 9–15 UTC, 4 November 2011. The locations of Prague and Budapest are marked. Modeled with the HYSPLIT model [10] using the GFS meteorological data.

Reports from Prague indicate clearly that the plume released on 4 November 2011 was also detected in the city in a concentration magnitude of 10 μBq/m^3^ [2]. However, preliminary dispersion simulations did not produce this result. In Lagrangian model simulations, the plume was predicted to move towards Eastern Czech Republic and Western Poland (Fig 4). In case of a larger release, it would have been extremely important to correctly predict whether or not the city of Prague could be reached by the plume, therefore this issue was investigated in details. Besides the trajectory models, the WRF-Chem atmospheric chemistry transport model was also used in this case to obtain a finer resolution and a more detailed weather and dispersion simulation to reveal the source of error. As it can be seen in Fig 4, WRF-Chem showed the northward and also a more north-westward motion of the plume, and correctly predicted its presence in Prague with a relatively high maximum one-hour mean concentration of 20 μBq/m^3^. Arrival time in Prague was 17 UTC, 4 November based on the WRF-Chem model result. The plume of HYSPLIT later marginally affected Prague between 0–6 UTC, 5 November on the very edge of the northward moving plume with peak concentration of 1.8 μBq/m^3^, a small value compared to the generally larger concentrations predicted by HYSPLIT (Fig 5). It is also remarkable that HYSPLIT dispersion and trajectory results were significantly different, warning of high uncertainties in transport directions (Fig 5).

**Fig 6:**
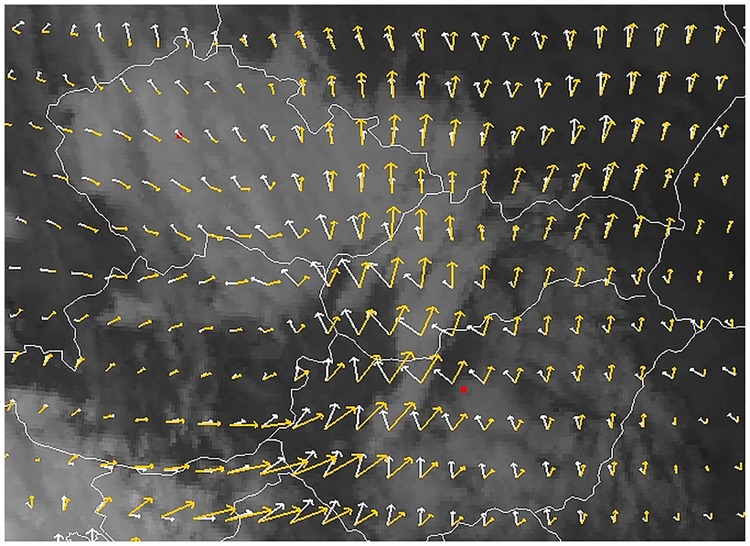
Visible satellite image at 12 UTC, 4 November 2011. Fog and stratus clouds covering the Czech Republic indicates the presence of a strong low-level inversion. Wind below the inversion layer (950 hPa, white arrows) differs significantly from the wind above the inversion layer (850 hPa, orange arrows). Red dots show the locations of Prague and Budapest. Data obtained from EUMETSAT and GFS.

The difference between model results can be understood by investigating the wind field on different levels. During the period of the release, a high pressure system caused cloudless weather in Eastern Europe, forcing the plume to spread northward on its rear edge. At the same time, low-level stratus clouds and fog covering almost the entire Czech Republic are well observable in the satellite image, indicating the presence of a strong inversion in the lower atmosphere, which is typical in late autumn anticyclones (Fig 6).

While the southerly wind transported the pollutants northward above the inversion, the near-surface wind turned towards Prague, which could cause the plume to reach the city (Fig 6). Meanwhile, in the middle and high troposphere, the wind above the eastern Czech Republic turned into north-western direction, flowing almost oppositely to the low-level flow.

The vertical wind shear explains the large difference among model results. The direction of transport largely depended on the height of the plume, the latter being very sensitive on turbulence parameterization and the PBL height data. Wind shear also reveals the reason behind the contradiction of HYSPLIT dispersion and trajectory results (Fig 5). Deterministic HYSPLIT trajectories were transported in lower altitudes than particles in the HYSPLIT dispersion simulation affected by strong stochastic vertical turbulent mixing. This separation in altitude led to different transport directions due to the vertical wind shear.

**Fig 7:**
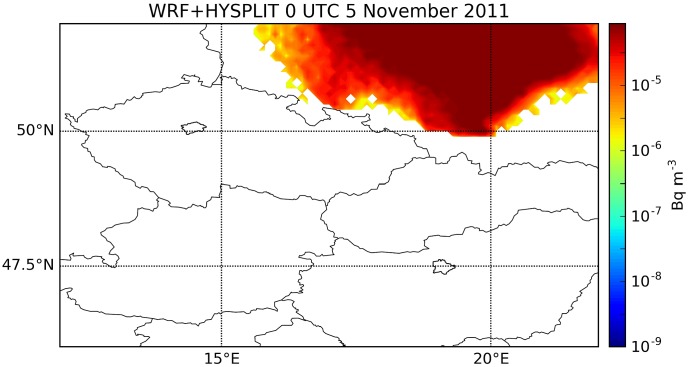
WRF-HYSPLIT model results. Surface concentration of ^131^I at 0 UTC, 5 November 2011 modeled with the HYSPLIT model [10] using the WRF meteorological data. The locations of Prague and Budapest are marked. Northward transport was caused by higher level winds.

To better understand the difference among model results, our WRF meteorological output was also used as an input of HYSPLIT trajectory model keeping the same dispersion settings as in the case of the GFS-driven simulation (Fig 7). Interestingly, the results showed a rapid northward transport, largely contradicting to what was obtained from WRF—Chem with the same meteorological data (Figs 4 and 7). Considering the wind field in Fig 6, we can assume that particles were lifted to higher altitudes in this simulation.

Besides the wind fields obtained from NWP model results (Fig 6), the radiosonde measurement in Prague at 18 UTC, 4 November 2011 also supports the assumption that the plume could only reach the city in the lowest layers. The wind changed with altitude from south-easterly to south-westerly in the layer between 750–1100 m above mean sea level, and turned further rightward with height to gain north-westerly direction above 2300 m. Therefore, the shear in wind direction exceeded 60° in the lowest 1100 m and 180° in the lowest 2400 m thick layer. The complexity of the weather situation was further strengthened by the fog and the inversion capping the near-surface plume.

## Discussion

It has been shown that although Lagrangian dispersion models provide reliable results in most cases, complex weather situations can give rise to prediction errors. Although mesoscale convection is usually considered to be a main source of uncertainty in atmospheric trajectories, the chance of errors occurring near a low-level inversion must not be underestimated. The detections in Dubna and Stockholm, both occurring under foggy conditions, warned that even a generally reliable dispersion simulation can show large uncertainties in surface concentrations if low-level inversion is present as the simulation of downward mixing of the upper-level plume is critical. Coinciding with vertical wind shear, the false prediction of vertical mixing can cause errors in the transport direction, an issue that has been demonstrated primarily through the case of a ^131^I release on 4 November, 2011 from the laboratory of the Institute of Isotopes Ltd., Budapest.

A low-level inversion with strong vertical wind shear was present over the Czech Republic. A significant portion of the emitted radioiodine was trapped below the inversion and transported by the low-level north-westward winds to Prague. This effect was poorly predicted in Lagrangian model simulations (RAPTOR and HYSPLIT). The source of this error can be identified in the high planetary boundary layer (PBL) heights provided by the meteorological model. Although the low-level wind shear at approximately 400–500 m altitude was correctly represented in the GFS meteorological data, the temperature inversion was underestimated and the PBL height was assumed to be 800–1200 m. Lagrangian models are designed to provide efficient mixing within the PBL, therefore simulated particles reached altitudes higher than the inversion in a large fraction of time. In reality, the inversion capped the low-level pollutants and provided very poor upward mixing. As a result, simulated particles followed a more eastern path, driven by winds above the inversion in a large fraction of the simulation time.

Lagrangian models show high sensitivity on the input PBL height data that can be uncertain in complex situations. The correct prediction of the WRF-Chem model can be explained by the online coupled simulation of meteorology and dispersion: instead of relying on turbulent mixing profiles fit to the overestimated PBL height, turbulent diffusion was online calculated at each vertical level within the primary model run. Indeed, the advantages of online coupling are not exclusive to the Eulerian approach and have also been shown in Lagrangian applications [28].

The significance of online coupling is further increased considering the fact that the overestimation of PBL heights was more serious in the WRF than in the GFS simulation, probably due to the finer representation of orography that further increased the vertical turbulence. The wind shear was large enough to cause 60–90° error in the transport direction by only a few hundred meters difference in the altitude of the plume. Therefore, HYSPLIT and RAPTOR model runs with WRF meteorology resulted in a rapid northward transport due to winds in the altitude between 1200–2000 m, a result that contradicts to the online coupled WRF-Chem simulation yielding slower northwestward plume motion by near-surface winds.

**Fig 8:**
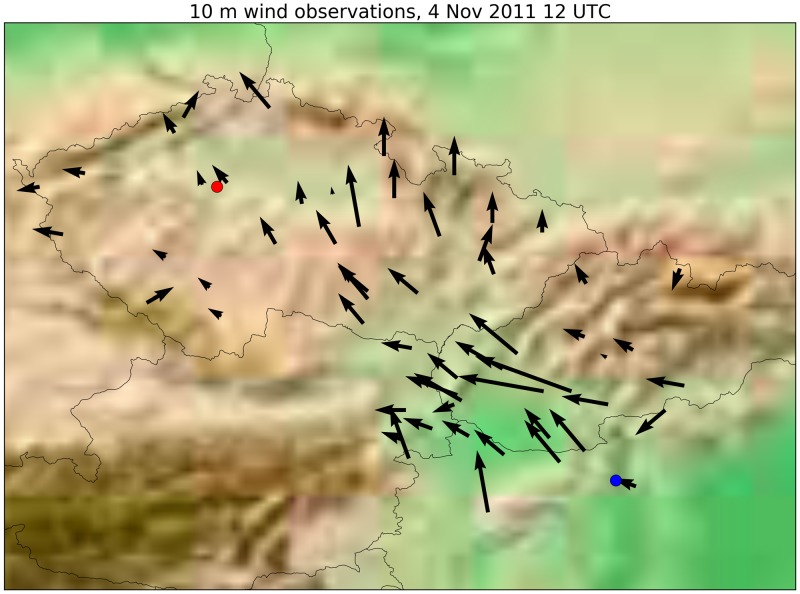
Surface wind observations at the time of the release, 12 UTC, 4 November 2011. Surface wind transporting the plume from Budapest to Prague is well observable. Data obtained from SYNOP reports of the National Weather Services of Austria, Slovakia, the Czech Republic and Hungary.

In real-life situations, it is usually not affordable to run WRF-Chem or another online integrated model in case of an accidental release. Although the transport of the plume to Prague had been poorly predicted by Lagrangian models, there were clues that would have made it possible to detect the error. Deterministic HYSPLIT trajectories showed a much more westward transport direction than dispersion results from the same model (Fig 5). It might have warned the user of the presence of the wind shear and a possibility of a significantly different pathway than the one predicted by the dispersion model. Furthermore, by simply looking at the surface wind observations during the release period, the chance of pollutants to reach Prague could have become obvious (Fig 8). These results underline the importance of correct interpretation of dispersion model results considering different types of atmospheric transport models and also basic meteorological aspects to identify possible error sources in dispersion model results.

## Conclusion

Atmospheric dispersion simulations have been carried out in cases of four 6-hour long ^131^I release from Budapest in October and November 2011. Model results from the Lagrangian dispersion model RAPTOR, developed at Eötvös Loránd University, were compared to those of Lagrangian HYSPLIT and Eulerian WRF-Chem, two of the most widely applied atmospheric transport models. Simulation results were evaluated based on reported detections in Dubna (Russia), Stockholm (Sweden) and Prague (Czech Republic). Dispersion models have been proven to provide reliable results on local to global scale in several studies and are essential tools of risk management. However, the users must not ignore possible error sources in complex weather situations characterized by low-level inversions with fog and a significant directional wind shear.

In three of the four investigated cases, the existence and the arrival time of the plume above the detection sites was well predicted. However, concentration values showed large uncertainty and variability among the models due to complex local weather with fog and very low mixing heights occurring in the two cities during the sampling period.

In case of the ^131^I detection in Prague, 4 November 2011, the simulated plume of Lagrangian models avoided or just marginally affected the city. On the other hand, the online coupled simulation of WRF-Chem showed the plume to largely move towards Prague. This contradiction was explained by a strong low-level wind shear and the overestimation of planetary boundary height in the meteorological models. We propose that possible errors in model results can be suspected without costly WRF-Chem simulations by considering deterministic trajectory calculations and surface wind observations. The results underline the importance of the identification of low-level inversions and wind shears in the interpretation of dispersion model results.

## References

[1] De Vismes Ott A, Masson O. Detection in France of trace levels of iodine isotopes from Hungary. ICRER 2014: 3 International Conference on Radioecology and Environmental Radioactivity; Barcelona (Spain) 2014.

[2] IAEA. Low Levels of Iodine Detected in Europe. Press Release 2011.

[3] Wotawa G. Source determination and localization by Atmospheric Transport Modelling. Symposium on International Safeguards; Vienna (Austria), IAEA; 2014.

[4] Reuters. Low levels of radioactive particles in Europe: IAEA. Reuters 2011.

[5] IAEA. Source of Iodine-131 in Europe Identified. Press Release 2011.

[6] Mészáros R, Leelőssy Á, Kovács T, Lagzi I. Predictability of the dispersion of Fukushima-derived radionuclides and their homogenization in the atmosphere. Sci Rep 2016;6. 10.1038/srep19915 PMC4730138 26817513

[7] Ryall DB, Maryon RH. Validation of the UK Met. Office’s NAME model against the ETEX dataset. Atmos Environ 1998;32:4265–4276.

[8] Stohl A, Hittenberger M, Wotawa G. Validation of the Lagrangian particle dispersion model FLEXPART against large-scale tracer experiment data. Atmos Environ 1998;32:4245–4264.

[9] Draxler RR, Hess GD. An overview of the HYSPLIT_4 modelling system for trajectories. Australian Meteorological Magazine 1998;47:295–308.

[10] Stein AF, Draxler RR, Rolph GD, Stunder BJB, Cohen MD, Ngan F. NOAA’s HYSPLIT Atmospheric Transport and Dispersion Modeling System. Bull Am Meteorol Soc 2015;96:2059–77.

[11] Leelőssy Á, Ludányi EL, Kohlmann M, Lagzi I, Mészáros R. Comparison of two Lagrangian dispersion models: a case study for the chemical accident in Rouen, January 21–22, 2013. Időjárás 2013;117:435–50.

[12] Leelőssy Á, Molnár F Jr, Izsák F, Havasi Á, Lagzi I, Mészáros R. Dispersion modeling of air pollutants in the atmosphere: a review. Central European Journal of Geosciences 2014;6:257–78.

[13] Moreira V, Degrazia GA, Robertia DR, Timm AU, da Costa Carvalho J. Employing a Lagrangian stochastic dispersion model and classical diffusion experiments to evaluate two turbulence parameterization schemes. Atmos Pollut Res 2011.

[14] Stohl A, Forster C, Frank A, Seibert P, Wotawa G. Technical note: The Lagrangian particle dispersion model FLEXPART version 6.2. Atmos Chem Phys 2005;5:2461–2474.

[15] Draxler R, Arnold D, Chino M, Galmarini S, Hort M, Jones A, et al World Meteorological Organization’s model simulations of the radionuclide dispersion and deposition from the Fukushima Daiichi nuclear power plant accident. J Environ Radioact 2015;139:172–84. doi: 10.1016/j.jenvrad.2013.09.014 24182910 10.1016/j.jenvrad.2013.09.014

[16] Koracin D, Vellore R, Lowenthal DH, Watson JG, Koracin J, McCord T, et al Regional Source Identification Using Lagrangian Stochastic Particle Dispersion and HYSPLIT Backward-Trajectory Models. J Air Waste Manag Assoc 2011;61:660–72. 21751582 10.3155/1047-3289.61.6.660

[17] McGowan H, Clark A. Identification of dust transport pathways from Lake Eyre, Australia using Hysplit. Atmos Environ 2008;42:6915–25.

[18] NOAA. NCEP GDAS/GFS Global Tropospheric Analyses and Forecast Grids 2016. http://www.emc.ncep.noaa.gov/GFS/ (accessed October 13, 2016).

[19] Grell GA, Peckham SE, Schmitz R, McKeen SA, Frost G, Skamarock WC, et al Fully coupled “online” chemistry within the WRF model. Atmos Environ 2005;39:6957–75.

[20] NOAA. WORKING GROUP 11: ATMOSPHERIC CHEMISTRY 2013. http://ruc.noaa.gov/wrf/WG11/ (accessed June 23, 2016).

[21] Baklanov A, Schlünzen K, Suppan P, Baldasano J, Brunner D, Aksoyoglu S, et al Online coupled regional meteorology chemistry models in Europe: current status and prospects. Atmos Chem Phys 2014;14:317–98.

[22] Jiang F, Wang T, Wang T, Xie M, Zhao H. Numerical modeling of a continuous photochemical pollution episode in Hong Kong using WRF—Chem. Atmos Environ 2008;42:8717–27.

[23] Tie X, Madronich S, Li G, Ying Z, Zhang R, Garcia AR, et al Characterizations of chemical oxidants in Mexico City: A regional chemical dynamical model (WRF-Chem) study. Atmos Environ 2007;41:1989–2008.

[24] Tuccella P, Curci G, Visconti G, Bessagnet B, Menut L, Park RJ. Modeling of gas and aerosol with WRF-Chem over Europe: Evaluation and sensitivity study. J Geophys Res 2012;117:D03303.

[25] Wang X, Liang X-Z, Jiang W, Tao Z, Wang JXL, Liu H, et al WRF-Chem simulation of East Asian air quality: Sensitivity to temporal and vertical emissions distributions. Atmos Environ 2010;44:660–9.

[26] Janjić ZI. Nonsingular implementation of the Mellor—Yamada level 2.5 scheme in the NCEP Meso model. NCEP Office Note 2002;437:61.

[27] Hu X-M, Doughty DC, Sanchez KJ, Joseph E, Fuentes JD. Ozone variability in the atmospheric boundary layer in Maryland and its implications for vertical transport model. Atmos Environ 2012;46:354–64.

[28] Ngan F, Stein A, Draxler R. Inline Coupling of WRF—HYSPLIT: Model Development and Evaluation Using Tracer Experiments. J Appl Meteor Climatol 2015;54:1162–76.

