# Correction: Numerical simulations of atmospheric dispersion of iodine-131 by different models

**DOI:** 10.1371/journal.pone.0178661

**Published:** 2017-05-23

**Authors:** The PLOS ONE Staff

There are errors in the funding section. The correct funding information is as follows: Support was provided by National Research, Development and Innovation Office of Hungary No. K116506 to I.L., the National Research, Development and Innovation Office of Hungary No. K109109 to R.M. and the New National Excellence Program of the Ministry of Human Capacities to Á.L. The publisher apologizes for the errors.
